# The impact of the Catechol-O-methyltransferase Val158Met polymorphism on survival in the general population – the HUNT study

**DOI:** 10.1186/1471-2350-8-34

**Published:** 2007-06-19

**Authors:** Knut Hagen, Lars J Stovner, Frank Skorpen, Elin Pettersen, John-Anker Zwart

**Affiliations:** 1 Department of Clinical Neuroscience, Faculty of medicine, Norwegian University of Science and Technology, Trondheim, Norway; 2 Norwegian National Headache Centre, Section of Neurology, St. Olavs Hospital, Trondheim, Norway; 3 Department of Laboratory Medicine, Children's and Women's health, Faculty of medicine, Norwegian University of Science and Technology, Trondheim, Norway; 4 National Centre for Spinal Disorders, St. Olavs Hospital, Trondheim, Norway

## Abstract

**Background:**

The catechol-*O*-methyltransferase (*COMT*) gene contains a functional polymorphism, Val158Met which has been related to common diseases like cancer, psychiatric illness and myocardial infarction. Whether the Val158Met polymorphism is associated with survival has not been evaluated in the general population. The aim of this prospective study was to evaluate the impact of codon 158 *COMT *gene polymorphism on survival in a population-based cohort.

**Methods:**

The sample comprised 2979 non-diabetic individuals who participated in the Nord-Trøndelag Health Study (HUNT) in the period 1995–97. The subjects were followed up with respect to mortality throughout year 2004.

**Results:**

212 men and 183 women died during the follow up. No association between codon 158 *COMT *gene polymorphism and survival was found. The unadjusted relative risk of death by non-ischemic heart diseases with Met/Met or Met/Val genotypes was 3.27 (95% confidence interval, 1.19–9.00) compared to Val/Val genotype. When we adjusted for age, gender, smoking, coffee intake and body mass index the relative risk decreased to 2.89 (95% confidence interval, 1.04–8.00).

**Conclusion:**

During 10 year of follow-up, the Val158Met polymorphism had no impact on survival in a general population. Difference in mortality rates from non-ischemic heart diseases may be incidental and should be evaluated in other studies.

## Background

The catechol-*O*-methyltransferase (*COMT*) gene located at chromosome 22q11.2 contains a functional polymorphism at codon 158 that has been the subject of several molecular epidemiological studies because of the important role of the COMT enzyme in the metabolism of catecholamines and catechol estrogens. A substitution of valine (Val) by methionine (Met) at codon 158 affects the activity of the COMT enzyme, and individuals with the Val/Val genotype have a three- to fourfold higher activity of the COMT enzyme than those with Met/Met genotype [1].

The Val158Met polymorphism has been linked to e.g. psychiatric disorders and pain perception [2-4], and several epidemiological studies have also reported an association to several potential fatal disorders. Presence of the Val/Val genotype has been considered to be favorable because it seems to lower the risk of developing non-Hodgkin lymphoma and estrogen-associated cancers in women [5-7], and because it is associated with a higher tendency to remain free from increase in prostate-specific antigen in men with prostate cancer [8]. Men with Val/Val genotype may also be less likely to commit suicide [9], and the Val allele has proved to be associated with less aggressive behaviors or violent suicide attempts in schizophrenic patients [10,11]. On the other hand, the Val/Val genotype seems to give a higher risk of myocardial infarction among hypertensive patients [12], and it has also been associated with high systolic blood pressure [13].

Despite the large number of studies evaluating the relationship between Val158Met polymorphism and diseases, the influence of this polymorphism on survival has not been evaluated. The aim of this prospective study was to evaluate the impact of codon 158 *COMT *gene polymorphism on survival in a population-based cohort.

## Results

The genotype distribution among the 2979 individuals (1347 men and 1632 women) was in Hardy-Weinberg equilibrium. The demographic data of the different genotype groups are shown in Table 2. The individuals in the three genotype groups did not differ significantly with regard to gender, age, body mass index, cholesterol level, smoking status, level of physical activity, coffee intake, prevalence of stroke, or education level. However, the prevalence of ischemic heart diseases (including angina pectoris and/or myocardial infarction) reported in connection with the HUNT study in 1995–97 tended to be lower among individuals with Val/Val genotype than among those with other genotypes (6.4 percent vs. 8.6 percent, *p *= 0.09). The individuals with known genotype were significantly older and had, accordingly, higher mean systolic and diastolic BP and higher mean cholesterol level than those without *COMT *data available (*p *< 0.05) (Table 2).

**Table 1:** Primers and hybridization probes used for COMT Val158Met genotyping

	Sequence
Primers:	Forward 5'-ACGCCGTGATTCAGGAGCA-3'
	Reverse 5'-GTCTTTCCTCAGCCCCAG-3'
Probes:	Sensor 5'-TCACGCCAGCGAAATCCA-Fl*-3'
	Anchor 5' LC Red 640^#^-ATCCGCTGGGTGATGGCG-3'

*Fl = Fluorescein

# LC Red 640 = Light Cycler Red 640

Underlined C indicates polymorphic site

**Table 2:** Clinical characteristics of the subjects according to *COMT *genotypes.

*Characteristics*	*No COMT genotyping (n = 62,247)*	*Met/Met (n = 951)*	*Met/Val (n = 1482)*	*Val/Val (n = 546)*
Sex, female (%)	53.2	54.0	55.1	55.1
Age, mean (SD)	49.1(18.9)^$^	52.8 (18.3)	52.7 (18.2)	52.1 (18.0)
Education > 12 years (%)	19.0	17.4	15.7	16.7
Mean systolic blood pressure (mmHg)(SD)	137.6 (22.3)^$^	140.9 (22.2)	140.4 (22.6)	141.6 (22.9)
Mean diastolic blood pressure (mmHg)(SD)	80.2 (12.5)^$^	81.7 (12.4)	81.0 (12.6)	82.3 (12.9)
Body mass index, kg/m^2 ^(SD)	26.4 (4.1)	26.3 (4.2)	26.4 (4.1)	26.3 (4.2)
Cholesterol, mmol (SD)	5.89(1.26)^$^	6.02 (1.31)	6.03 (1.30)	6.08 (1.37)
Current smoking (%)	29.0	28.0	29.6	29.0
High level of physical activity* (%)	24.1	19.9	18.7	20.1
Coffee intake > 6 cups/day (%)	20.1	19.6	18.6	19.0
Use of antihypertensive medication (%)	11.0	12.6	13.3	11.2
Myocardial infarction (%)	3.3	3.7	3.5	2.8
Angina pectoris (%)	5.0	6.9	7.1	5.9
Stroke (%)	1.9	2.5	1.6	1.8

* High level of physical activity was defined as high intensity exercise more than 1 hour per week.

$ *p *< 0.05

### Overall mortality

**Figure 1:**
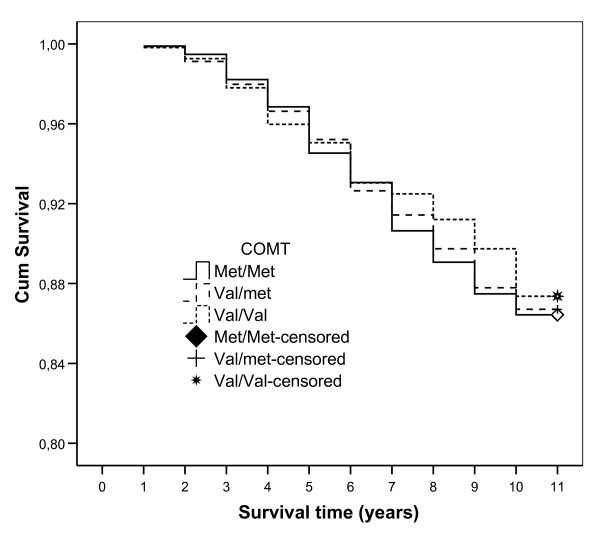
Survival curves for different genotypes. Log-rank test: *p *= 0.87.

212 (15.7 percent) men and 183 (11.2 percent) women died during the follow up (*p *< 0.001). The distribution of the COMT Val158Met alleles was similar among survivors and the dead (Table 3), evident for both genders (data not shown). No difference in survival was found for the different genotypes (Fig. 1).

**Table 3:** Mortality from various causes by January 1, 2005 related to *COMT *genotypes and 5- and 10-year survival.

	*Met/Met (n = 951)*		*Met/Val (n = 1482)*		*Val/Val (n = 546)*		*5-year survival*	*10-year survival*
	n	%	n	%	n	%	p*	p*
Genotypes								
Total deaths	129	32.7	197	49.8	69	17.5	0.77	0.87
Neoplasms (C00-D48)	35	33.3	52	49.5	18	17.1	0.97	0.93
Cardiovascular causes (I00-I99)	58	31.9	92	50.5	32	17.6	0.47	0.96
All other causes	36	33.3	53	49.1	19	17.6	0.72	0.94
								
Alleles	Met		Val					
Total deaths	455	57.6	335	42.4			0.35	0.63
Neoplasms (C00-D48)	122	58.1	88	41.9			0.74	0.48
Cardiovascular causes (I00-I99)	208	57.1	156	42.9			0.30	0.57
All other causes	125	57.9	91	42.1			0.98	0.51

* p value for the log-rank test

### Death caused by neoplasms (ICD-10 C00-D48)

61 men (4.5 percent) and 44 women (2.7 percent) (*p *= 0.007) had neoplasm as the underlying cause of death. No difference in cancer mortality was found between the different Val158Met genotype groups (Table 4). Cancer in the digestive system (C00-C26) was common among women (39 percent), whereas 20 men (33 percent) died because of prostate cancer (C61). Slightly more men with Val/Val genotype had prostate cancer as *underlying *cause of death than those with other genotypes (2.0 percent versus 1.4 percent, *p *= 0.44, long-rank test). However, no such tendency was found when including 8 men with prostate cancer as *immediate *or *contributing *cause of death (2.0 percent versus 2.0 percent, *p *= 0.95, log-rank test)

**Table 4:** Causes of death according to *COMT *genotypes

	*Met/Met (n = 951)*		*Met/Val (n = 1482)*		*Val/Val (n = 546)*		*Val/Val vs. other genotypes*
	n	%	n	%	n	%	Log-rank p value
Neoplasms (C00-D48)	35	3.7	52	3.5	18	3.3	0.74
Cancers in the digestive system (C00-C26)	14	1.5	14	0.9	4	0.7	0.39
Cancer in the respiratory system (C30-C39)	6	0.6	11	0.7	1	0.2	0.23
Breast cancer/cancer in female genital organs (C50-C58)	2	0.2	2	0.1	1	0.2	1.00
Prostate cancer (C61)	5	0.5	10	0.7	6	1.1	0.24
All other neoplasms	8	0.8	15	1.0	6	1.1	0.92
Cardiovascular causes (I00-I99)	58	6.1	92	6.2	32	5.9	0.77
Ischemic heart diseases (I20-I25)	29	3.0	43	2.9	15	2.7	0.78
Non-ischemic heart diseases (I30-I33 and I39-I52)^#^	12	1.3	20	1.3	2	0.4	0.06
Cerebrovascular diseases (I60-I69)	10	1.1	19	1.3	9	1.6	0.39
All other vascular deaths	7	0.7	10	0.7	6	1.1	0.33
Sudden death (R96)	1	0.1	3	0.2	1	0.2	0.92
Accidents (V01-V99, W00-W99, and X00-X99)	7	0.7	10	0.7	5	0.9	0.87
Dementia (F03, G30, and R54)	7	0.7	6	0.4	1	0.2	0.49
Diseases of the respiratory system (J00-J99)	12	1.3	13	0.9	5	0.9	0.95
Diseases of the digestive system (K00-K93)	3	0.3	7	0.5	4	0.7	0.33
Diseases of the kidney and ureter (N00-99)	3	0.3	5	0.3	0	0	0.31
Other diseases*	3	0.3	9	0.6	3	0.5	0.87

# Non-ischemic heart diseases (I30-I33 and I39-I52): *Underlying *cause of death among 34 individuals.

Additionally, *immediate *cause of death among 28 others (in total 62 individuals)

* Infections (A00-B99), diseases of the nervous system (G00-G99), mental and behavioral disorders (F00-F99), and other symptoms, sign, abnormal findings, ill-defined causes (R00-R94).

### Cardiovascular causes (ICD-10 I00-I00)

**Figure 2:**
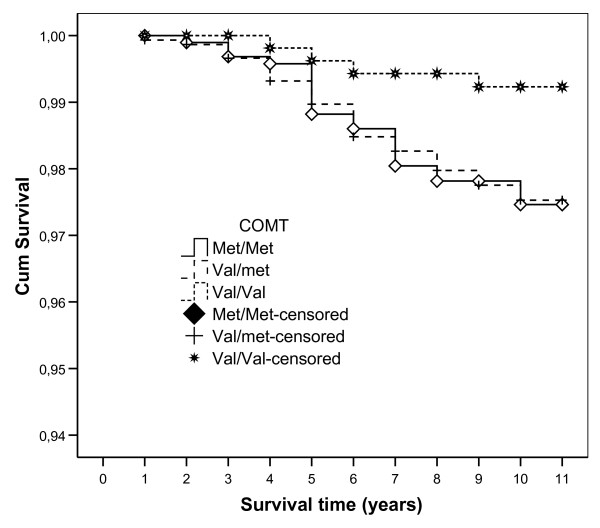
Survival curves for different genotypes considering only death from non-ischemic heart diseases (I30-I33, I39-I52). Log-rank test for Val/Val versus other genotypes: *p *= 0.015.

Cardiovascular disease was the *underlying *cause of death in 97 men (7.2 percent) and 85 women (5.2 percent) (*p *= 0.02). Among these, 87 individuals died of ischemic heart disease (I20-I25) and 34 of non-ischemic heart diseases (I30-I33 or I39-I52) (Table 4). When the Met/Met and Val/Met genotypes were pooled, a borderline significant tendency to lower mortality caused by non-ischemic heart diseases was found among individuals with Val/Val genotype (n = 2) compared to those with other genotypes (n = 32) (*p *= 0.06, rank-log test). Among the 62 individuals with non-ischemic heart diseases as *underlying *(n = 34) or *immediate *cause (n = 28) of death, subjects with Val/Val had lower mortality rates (n = 4) than those with the Met/Val or Met/Met genotypes (n = 58) (*p *= 0.015, rank-log test) (Figure 2). The unadjusted relative risk of death by non-ischemic heart diseases with Met/Met or Met/Val genotypes was 3.27 (95% confidence interval, 1.19–9.00) compared to Val/Val genotype. When we adjusted for age, gender, smoking, coffee intake and body mass index the relative risk decreased to 2.89 (95% confidence interval, 1.04–8.00).

### Mortality among 69 with self-reported diabetes mellitus

During the 10 year follow up the overall mortality was more than three times higher among the excluded 69 individuals with self-reported diabetes mellitus than among the included sample of 2979 non-diabetic subjects (47.8% versus 13.3%, p < 0.001). However, our results were not substantially changed when we repeated the analyses including these 69 diabetic persons. Specifically, out of the 33 diabetic who died during the follow up, a total of 6 had non-ischemic heart diseases as *underlying *(n = 3) or *immediate *cause (n = 3) of death. Even when these were included, subjects with Val/Val had lower mortality rates (n = 6) than those with the Met/Val or Met/Met genotypes (n = 62) (*p *= 0.04, rank-log test).

## Discussion

In this population-based sample of individuals the Val158Met polymorphism at the *COMT *gene had no impact on survival in general.

The strength of this study was the prospective cohort design which provides a valuable complement to case-control studies and with ability to adjust for potential confounding factors [19]. Furthermore, the *COMT *genotyping was performed in a random sample of individuals drawn from a genetically homogenous white Norwegian population and being unselected except from the fact that they did not have diabetes. Less than 3 percent of subjects were of non-white ethnicity. Bias caused by admixed ethnicities may be reduced in population-based studies when a genetically homogenous population is investigated.

The majority of included participants were selected completely at random, but were mixed together with a non-diabetic group that were age-matched controls for the purpose of a different genetic study on diabetes. It would have been preferable to perform the analyses on a completely random selected population, including both diabetic and non-diabetic persons, but this strategy was not possible because of the format of the data file. Because diabetes status was a selection criterion for approximately 30 percent of the sample, we decided to exclude 69 individuals with diabetes mellitus, making the whole group more homogenous. Individuals with Val/Val seem to be less likely to be diabetic [16]. If true, because of a high mortality rate among those with diabetes, the Val/Val genotype should be associated with lower mortality rates. On the other hand, one might speculatively assume that diabetic individuals with Val/Val genotype have a less favorable prognosis and higher mortality than diabetics with other genotypes. Thus, how the Val158Met polymorphism influences on mortality among persons with diabetes mellitus is difficult to predict, but our exclusion of the 69 persons with self-reported diabetes probably minimize the risk of bias. However, the main results were not substantially changed when the analyses were repeated including these 69 diabetic persons.

Our sample of 2979 individuals should have enough power to detect a difference in survival between genotypes that is of clinical interest, even when considering smaller subgroups deceased because of neoplasms or heart diseases. However, the number of individuals who died from accidents, suicide, prostate cancer, non-Hodgkin lymphoma, breast cancer or cancer in female genital organs was very low. For such relatively rare causes of death the impact of Val158Met polymorphism needs to be evaluated with other study designs. An example of this is a prospective study from United Kingdom of 2430 women with breast cancer reporting no effect on survival from polymorphisms in the COMT gene [20].

In the present study no association between overall cardiovascular mortality and Val158Met polymorphism was found. However, when the Met/Met and Val/Met genotypes were pooled, individuals with Val/Val genotype tended to be less likely to die because of heart diseases classified as I30-I33 or I39-I52 compared to those with other genotypes, and the difference was significant even when we controlled for the most important confounding factors. It should be noted that this result may be due to chance because of the multiple hypotheses being tested. Hence, the association between the Val/Val genotype and death from non-ischemic heart disease should be confirmed in a lager study.

Val/Val genotype is associated with high COMT enzyme activity, and speculatively, rapid metabolism of circulating catecholamines may be protective for some type of heart diseases e.g. the arrhythmias. Theoretically, haplotypes of the COMT gene may influence COMT activity. However, because of lack of financial and personnel resources we did not evaluated other single nucleotide polymorphisms within the COMT gene or in other genes, and as a consequence, we did not have the opportunity to evaluate haplotypes of the COMT gene or possible interaction with other genes.

Few other studies have evaluated the influence of COMT genotypes on heart diseases. In accordance with our study, in a Finnish cohort of 773 men, Met/Met genotype and heavy coffee intake was associated with higher incidence of acute coronary events than those with other genotypes [16]. In contrast to our findings, a Swedish study found the Met/Met genotype to be associated with decreased risk of myocardial infarction, but this study was performed in a highly selected group of hypertensive individuals [12].

## Conclusion

In this population-based study evaluating the impact of the COMT Val158Met polymorphism on survival among 2,979 adults, the polymorphism had no influence on total mortality.

## Methods

### Study population

Between August 1995 and June 1997, all inhabitants aged 20 years or older in Nord-Trøndelag county in Norway (n = 92,936) were invited to participate in the Nord-Trøndelag Health Survey ("Helseundersøkelsen i Nord-Trøndelag" = HUNT). In brief, two questionnaires including more than 200 health-related questions were administrated to the participants. The population in Nord-Trøndelag County was ethnically homogeneous (less than 3 percent of subjects were of non-white ethnicity), making it suitable for epidemiological genetic research [14].

Out of the 92,936 invited individuals, a total of 65,291 subjects (70 percent) answered the first questionnaire and participated in the health examination. Details of the non-participants are described elsewhere [14,15].

In the HUNT 2 biobank a total of 60,241 DNA samples were available. Genotyping of the *COMT *locus was performed among 3048 individuals. Approximately 70 percent were selected completely at random, whereas the remaining 30 percent included had been randomly selected among an *older *group of individuals who did not have self-reported diabetes mellitus. This latter group was generated in connection with a planned genetic study on diabetes that needed *age-matched *controls to a diabetic population. In the present sample the prevalence of self-reported diabetes mellitus was slightly lower among individuals with Val/Val genotype than the other genotypes (2.0% versus 2.3%, p = 0.61), and also in a Finnish study individuals with Val/Val were less likely to be diabetic [16]. In the unidentified data file all participants were mixed, and separate analyses of the group selected completely by random was not possible. Thus, with intention to have a homogeneous study cohort, those with self-reported diabetes (n = 69) in the randomly selected group were eliminated [13], resulting in a population of 2979 individuals for the final analysis. All age groups were included, but because the age-matched controls to a diabetic population as a group are older than the general population, the total group of 2979 individuals without self-reported diabetes was 3.5 years older (mean 52.6 years versus 49.1 years) than the HUNT population as a whole.

### Causes of death

We performed mortality follow-up by record linkage using the Norwegian 11-digit birth number (date of birth plus a 5-digit identifier), which is unique to each person residing in Norway, to obtain the date and underlying cause of death kept by Statistics Norway. The HUNT data including genotyping at the COMT locus were linked to the National Cause of Death register, and our report is based on mortality follow-up through the year 2004. We classified the 395 deaths on the basis of the *underlying *cause of death coded at Statistics Norway by using the 9th (death before 1996), or 10th revisions of the International Classification of Diseases (ICD). To further explore the influence of the Val158Met polymorphism on mortality from cancer and heart diseases on death, we also evaluated the *immediate *cause of death and up to five *contributing *causes of death. The *underlying *cause of death was defined as: a) the disease or injury that initiated the chain of morbid events leading directly to death or b) the external circumstances of the accident or violence that was the cause of the fatal injury [17]. By *immediate *cause of death was meant the disease, injury or condition directly leading to death and which was caused by the underlying cause of death [17]. *Contributing *cause of death was meant the condition that may have contributed to the death, but was not the direct causal relation with the disease or condition that has caused the death [17].

### Genotyping of the COMT locus

Blood sampling was done whenever subjects attended, and details for the procedure and the HUNT 2 biobank are described elsewhere [14]. DNA for genotyping was extracted from peripheral blood leukocytes from whole blood or blood clots stored in the HUNT 2 biobank, using the Puregene kit (Gentra Systems Inc.) manually or with an Autopure LS (Gentra Systems Inc.). Laboratory technicians were blinded to the data from the National Cause of Death register. *COMT *genotypes were determined using the LightCycler (Roche Diagnostics Scandinavia AB, Bromma, Sweden) fluorescence resonance energy transfer method [18]. Polymerase chain reaction (PCR) amplifications were performed in 20 μl reactions on a LightCycler System, using 2 μl genomic DNA and the LightCycler-FastStart DNA Master Hybridization Probes kit (Roche Diagnostics Scandinavia AB, Bromma, Sweden). PCR primers (Eurogentec, Seraing, Belgium) and fluorescence labeled probes (PROLIGO, Paris, France) are shown in Table 1. Based on melting curve profiles, participants were classified as having Val/Val, Val/Met, or Met/Met genotypes. Details on PCR and melting curve conditions are available on request.

### Ethics

The study was approved by the Regional Committee for Ethics in Medical Research, by the Norwegian Data Inspectorate, and by the Directorate for Health and Social Affairs.

### Statistical analysis

Differences between continuous variables were tested with analyses of variance (one-way ANOVA) and between dichotomous variables with the chi-square test (including Fisher's exact test). Analyses used two-tailed estimation of significance, and p < 0.05 was considered to be statistically significant. A Kaplan-Meier method was performed to estimate the cumulative survival and differences between genotypes were tested with a log rank test. We used the Cox proportional hazards model to adjust for age, gender and other potential confounding factors, including smoking status, level of education, use of alcohol, blood pressure, coffee intake and body mass index.

Overall, our sample of 2979 individuals had more than 80 percent power to detect a 3 percent difference in total mortality and mortality by cancer or heart diseases between pooled genotypes with 95 percent certainty.

Statistical analyses were performed using the Statistical Package for the Social Sciences (SPSS), version 14.0 (SPSS Inc, Chicago).

## Abbreviations

COMT = Catechol-*O*-methyltransferase

HUNT = "Helseundersøkelsen i Nord-Trøndelag" (The Nord-Trøndelag Health Study)

Val = Valine

Met = Methionine

ICD = International Classification of Diseases

## Competing interests

The author(s) declare that they have no competing interests.

## Authors' contributions

KH conceived of the study and performed the statistical analysis. KH, LJS, FS, and JAZ all participated in the design and drafted the manuscript. EP carried out the genotyping. All authors read and approved the final manuscript.

## Pre-publication history

The pre-publication history for this paper can be accessed here:


